# Altered *Slc25* family gene expression as markers of mitochondrial dysfunction in brain regions under experimental mixed anxiety/depression-like disorder

**DOI:** 10.1186/s12868-018-0480-6

**Published:** 2018-12-11

**Authors:** Vladimir N. Babenko, Dmitry A. Smagin, Anna G. Galyamina, Irina L. Kovalenko, Natalia N. Kudryavtseva

**Affiliations:** 1 Laboratory of Neuropathology Modeling, The Federal Research Center Institute of Cytology and Genetics SB RAS, Novosibirsk, Russia; 2 Neurogenetics of Social Behavior Sector, The Federal Research Center Institute of Cytology and Genetics SB RAS, Novosibirsk, Russia; 3 Laboratory of Human Molecular Genetics, The Federal Research Center Institute of Cytology and Genetics SB RAS, Novosibirsk, Russia

**Keywords:** RNA-Seq, Social defeat stress, Depression, Aggression, Slc25a* genes, Mrp* genes, Brain regions

## Abstract

**Background:**

Development of anxiety- and depression-like states under chronic social defeat stress in mice has been shown by many experimental studies. In this article, the differentially expressed Slc25* family genes encoding mitochondrial carrier proteins were analyzed in the brain of depressive (defeated) mice versus aggressive mice winning in everyday social confrontations. The collected samples of brain regions were sequenced at JSC Genoanalytica (http://genoanalytica.ru/, Moscow, Russia).

**Results:**

Changes in the expression of the 20 Slc25* genes in the male mice were brain region- and social experience (positive or negative)-specific. In particular, most Slc25* genes were up-regulated in the hypothalamus of defeated and aggressive mice and in the hippocampus of defeated mice. In the striatum of defeated mice and in the ventral tegmental area of aggressive mice expression of mitochondrial transporter genes changed specifically. Significant correlations between expression of most Slc25* genes and mitochondrial *Mrps* and *Mrpl* genes were found in the brain regions.

**Conclusion:**

Altered expression of the Slc25* genes may serve as a marker of mitochondrial dysfunction in brain, which accompanies the development of many neurological and psychoemotional disorders.

**Electronic supplementary material:**

The online version of this article (10.1186/s12868-018-0480-6) contains supplementary material, which is available to authorized users.

## Background

Mitochondrial dysfunction associated with mutations of one or more mitochondrial genes is thought to be involved in neurodegenerative disorders [1, 2] such as amyotrophic lateral sclerosis, Leigh’s syndrome, multiple sclerosis etc. It is suggested that mitochondrial dysfunction of several genes regulating mitochondrial function, morphology, and dynamics [3] plays an early and preponderant role in the pathogenesis of Alzheimer’s disease [4, 5] and Parkinson’s disease [6]. Growing evidences indicate that mitochondrial dysfunction may also be involved in the pathophysiology of schizophrenia, autism and affective spectrum disorders and others [7–12]. In spite of domination of the theory that a depletion in the levels of monoamines, including serotonin, is a trigger of depression [13, 14], the publications of last years strongly support the negative impact of mitochondrial dysfunction on synaptic plasticity and neurogenesis in depression [11, 15–17]. The concepts of mitochondrial dysfunction and monoamines are thought to be interrelated [9] and mitochondrial dysfunction is considered ubiquitous to many psychiatric disorders, including bipolar disorder and depression [18–23].

In our study we use the model of depression induced by chronic social defeat stress [24], which has been widely accepted in the original version and with modifications [24–26]. This rodent model satisfies all criteria suggested for a relevant model of depression [27]: etiology, symptomatology, sensitivity to antidepressants and anxiolytics treatments. Neurochemical changes in the brain are similar to those in humans [28, 29]. It has also been shown that the development of mixed anxiety/depression-like states in repeatedly defeated mice are accompanied by numerous molecular changes in the brain [25, 30–33]. Analysis of brain genomic changes in depressive mice in comparison with the mice with alternative social experience, i.e. chronic aggression leading to the development of behavioral pathology similar to psychosis [34, 35], enabled us to reveal specific and nonspecific changes in the brain regions of affected mice. A 21-day period of agonistic interactions is accompanied by changes in serotonin metabolism, serotonergic gene expression [28, 32, 36, 37] and expression of mitochondrial Mrpl* and Mrps* genes [33, 38] in different brain regions of mice, indicative of possible mitoribosomal biogenesis abnormalities. To confirm this observation, using the same RNA-Seq database we analyzed differential expression of mtSlc25a* genes encoding mitochondrial carriers from a superfamily of nucleus-encoded proteins that have been localized mostly at the inner mitochondrial membrane and serve as transporters of numerous metabolites, nucleotides, cofactors and inorganic anions [39] in the brain of chronically defeated mice and aggressive mice. We also studied correlations between FPKM levels of differentially expressed mitochondrial transporter family Slc25a* genes and mitoribosomal Mrpl* and Mrps* genes in different brain regions.

Detailed description of mitochondrial proteins and genes as well as their functions and dysfunctions is presented by Palmieri and co-authors [1, 40–42]. We assume that this study may be useful for understanding the mechanisms of mitochondrial dysfunction during development of depression and for search of the ways of possible pharmacologic correction.

## Methods

### Animals

Adult C57BL/6 male mice were obtained from Animal Breeding Facility, Branch of Institute of Bioorganic Chemistry of the RAS (Pushchino, Moscow region). Animals were housed under standard conditions (at a constant temperature of 22 ± 2 °C, 12:12 h light/dark regime starting at 8:00 am, with food in pellets and water available ad libitum). Mice were weaned at 3 weeks of age and housed in groups of 8–10 in standard plastic cages. Experiments were performed with 10–12 week old animals. All procedures were in compliance with the European Communities Council Directive 210/63/EU on September 22, 2010. The study was approved by Scientific Council No9 of the Institute of Cytology and Genetics SD RAS of March, 24, 2010, N 613 (Novosibirsk, http://spf.bionet.nsc.ru/).

### Generation of alternative social behaviors under agonistic interactions in male mice

Repeated positive and negative social experience, wins and defeats, in male mice were induced by daily agonistic interactions [24, 43]. Pairs of animals were each placed in a cage (14 × 28 × 10 cm) bisected by a transparent perforated partition allowing the animals to hear, see and smell each other, but preventing physical contact. The animals were left undisturbed for 2 days to adapt to new housing conditions and sensory contact before they were exposed to agonistic encounters. Every afternoon (14:00–17:00 p.m. local time) the cage cover was replaced by a transparent one, and 5 min later (the period necessary for activation), the partition was removed for 10 min to encourage agonistic interactions. The superiority of one of the mice was established within two or three encounters with the same opponent. The superior mouse would be chasing, biting and attacking another, who would be demonstrating only defensive behavior (upright or sideways postures, withdrawal etc.). Aggressive interactions between males are discontinued by lowering the partition if the strong attacking behavior has lasted 3 min (in some cases less) preventing the damage of defeated mice. Each defeated mouse (loser, defeater) was exposed to the same winner for 3 days, while afterwards each loser was placed, after the fight, in an unfamiliar cage with an unfamiliar winning partner behind the partition. Each aggressive mouse (winners), remained in its own cage. This procedure was performed once a day for 20 days and yielded an equal number of the losers and winners.

Three groups of animals were used: (1) Controls—mice without a consecutive experience of agonistic interactions; (2) Losers—chronically defeated mice; (3) Winners—chronically aggressive mice. The losers and winners with the most expressed behavioral phenotypes were selected for the transcriptome analysis. The winners demonstrated the biggest number and total attacking time and shortest latency of first attack, as well as aggressive grooming, threats (tail rattling), hostility during 20 day experiment. The losers showed full submission (posture “on the back”), avoidance, or the biggest time of passive defense (freezing, immobility) during the all time of the agonistic interaction test. The control animals and the affected mice, 24 h after the last agonistic interaction, were simultaneously decapitated. The brain regions were dissected by the same experimenter according to the Allen Mouse Brain Atlas map [http://mouse.brain-map.org/static/atlas]. All biological samples were placed in RNAlater solution (Life Technologies, USA) and were stored at − 70 °C until sequencing.

The brain regions selected for the analysis based on their functions and location of the neurons of neurotransmitter systems were as follows: the midbrain raphe nuclei, a multifunctional region of brain containing the body of the serotonergic neurons; the ventral tegmental area (VTA) containing the pericaryons of the dopaminergic neurons, which are widely implicated in brain reward circuitry and are important for motivation, cognition, drug addiction, and emotions relating to several psychiatric disorders; the striatum, which is responsible for the regulation of stereotypical behaviors and motor activity and is also involved in cognitive processes; the hippocampus, which belongs to the limbic system and is essential for memory consolidation and storage and plays an important role in emotional mechanisms and neurogenesis; the hypothalamus, which regulates stress reaction and many physiological processes.

### RNA-Seq

**Fig. 1:**
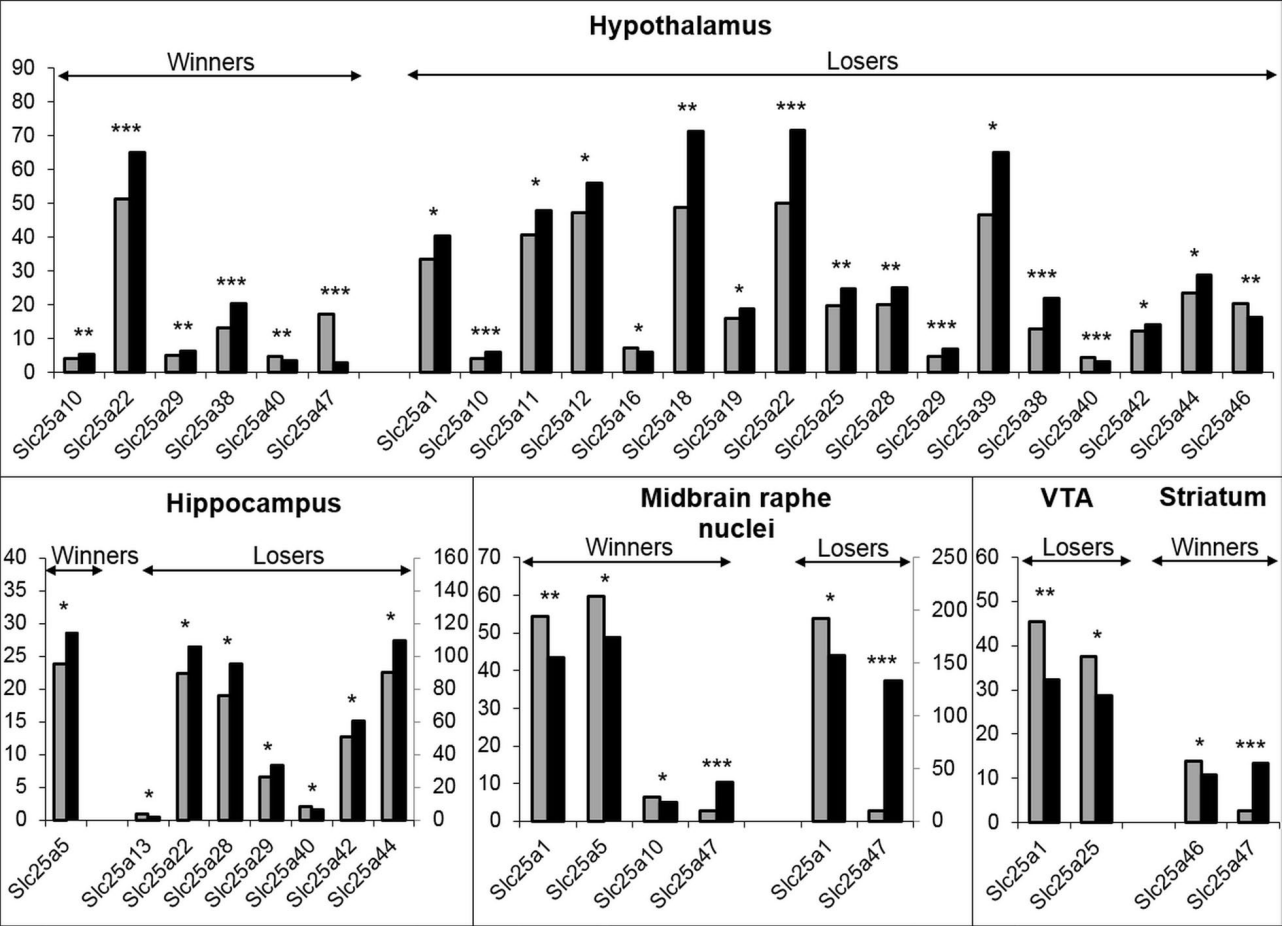
Differentially expressed Slc25a* genes in the brain regions in mice with different social experience. Winners—aggressive mice with repeated experience of aggression; Losers—defeated mice in daily agonistic interactions. Grey columns—controls, black columns—winners or losers. *p < .05; **p < .01; ***p < .001

We used the methods described earlier [33, 38]. The collected samples were sequenced at JSC Genoanalytica (www.genoanalytica.ru, Moscow, Russia), and the mRNA was extracted using a Dynabeads mRNA Purification Kit (Ambion, Thermo Fisher Scientific, Waltham, MA, USA). cDNA libraries were constructed using the NEBNext mRNA Library PrepReagent Set for Illumina (New England Biolabs, Ipswich, MA USA) following the manufacturer’s protocol and were subjected to Illumina sequencing. More than 20 million reads were obtained for each sample. The resulting “fastq” format files were used to align all reads to the GRCm38.p3 reference genome using the TopHat aligner [44]. DAVID Bioinformatics Resources 6.7 (http://david.abcc.ncifcrf.gov) was used for the description of differentially expressed gene ontology. The Cufflinks program was used to estimate the gene expression levels in FPKM (fragments per kilobase of transcript per million mapped reads) units and subsequently identify the differentially expressed genes in the analyzed and control groups. Detailed description of statistics for differentially expressed Slc25a* genes in brain regions is presented in Fig. 1, and in Additional file 1: Tables S1–S3. Each brain area was considered separately for 3 versus 3 animals. Genes were considered differentially expressed at *p *≤ .05 and corrected for multiple comparisons at *q* < .05.

We have previously conducted studies of gene expression in males in similar experiments using the RT-PCR method with a larger number of samples for each compared experimental group, i.e., winners and losers (> 10 animals). The direction and extent of changes *vs.* control in the expression of the *Tph2, Slc6a4, Bdnf, Creb1*, and *Gapdh* genes in the midbrain raphe nuclei of males as determined by the two methods, RT-PCR [36, 37] and RNA-Seq [32], are generally consistent. In order to cross-validate the results obtained, we employed the unique resource from Stanford University, USA [45] and found a significant concordance with our RNA-Seq data pool [46]. These findings suggest that the transcriptome analyses of the data provided by the JSC Genoanalitika (http://genoanalytica.ru, Moscow) have been verified and that the method reflects the actual processes that occur in the brain under our experimental paradigm. The Human Gene Database (http://www.genecards.org/); Online Mendelian Inheritance in Man database (http://omim.org/); Human disease database (MalaCards, http://www.malacards.org) were used for the description and analysis of the data obtained.

### Statistical analysis

For the transcriptome data, a Principal Component Analysis **(**PCA) was conducted using the XLStat software package (www.xlstat.com). PCA was based on Pearson product moment correlation matrix calculated on the FPKM value profiles of 47 analyzed genes. We also used a Pearson correlation as a similarity metric for the Agglomerative Hierarchical Clustering (AHC) and MultiDimentional Scaling (MDS). The agglomeration method comprised an unweighted pair-group average. The identification of alternatively spliced events in the RNA-Seq data was performed with rMATs software. Refseq v. 10.0 was used as a template for mapping the reads and alternative event annotations. Only exon skipping events were considered.

## Results

Different changes in the expression of 20 Slc25a* family genes in the hypothalamus, midbrain raphe nuclei, hippocampus, ventral tegmental area (VTA), and striatum were found in the male mice with alternative social experience (Fig. 1; Additional file 1: Table S1). Most differentially expressed Slc25a* genes were found in the hypothalamus (17 genes in defeated mice and 6 genes in aggressive mice), hippocampus (7 and 1 genes, respectively), and the midbrain raphe nuclei (2 and 4 genes, respectively). The smallest number of differentially expressed Slc25a* genes were found in the striatum—2 genes in aggressive mice and 2 genes in the VTA of defeated mice. Most differentially expressed Slc25a* genes in the hypothalamus of defeated and aggressive mice as well as in the hippocampus of defeated mice were up-regulated. In the VTA the Slc25a* genes changed expression in defeated mice and in the striatum of aggressive mice. The full list of data expressed in FPKM units for the Slc25a* genes in different brain regions of the mice of both social groups is presented in Additional file 2: Table S1.

In the hypothalamus (Fig. 1) the Slc25a* genes increased their expression in aggressive and depressive mice in comparison with the controls: *Slc25a10* (*p* < .007 and *p* < .0001; *q* < .002, respectively)*, Slc25a22* (*p* < .0006; *q* ≤ .018 and *p* < .0001; *q* < .001, respectively)*, Slc25a29* (*p* ≤ .011 and *p* < .0011; *q* < .010 respectively)*, Slc25a38* (*p *< .0001; *q *< .0034 and *p* < .0001; *q* < .001, respectively), and decreased expression of the *Slc25a40* (*p* < .0001; *q* ≤ .003, and *p* < .001; *q* ≤ .001, respectively) as well as *Slc25a47* (*p* < .0001; *q* ≤ .003) genes in aggressive mice. In defeated mice expression of *Slc25a1* (*p* < .017), *Slc25a11* (*p* < .030), *Slc25a12* (*p* < .032), *Slc25a18* (*p* ≤ .007; *q* < .038), *Slc25a19* (*p* < .030), *Slc25a25* (*p* < .004; *q* < .025), *Slc25a28* (*p *≤ .009; *q* < .046), *Slc25a39* (*p* < .021), *Slc25a42* (*p* < .038), *Slc25a44* (*p* < .014) increased and expression of *Slc25a16* (*p* < .019), *Slc25a46* (*p* < .003; *q* < .020) decreased.

In the hippocampus (Fig. 1) expression of the *Slc25a5* gene increased in aggressive mice (*p* < .029) as compared to the controls. In the defeaters expression of the *Slc25a22* (*p* ≤ .028), *Slc25a28* (*p* < .019), *Slc25a29* (*p* < .034), *Slc25a42* (*p* ≤ .044), *Slc25a44* (*p *≤ .022) genes increased and expression of the *Slc25a13* (*p* ≤ .011) and *Slc25a40* (*p* ≤ .036) genes decreased.

In the midbrain raphe nuclei (Fig. 1) decreased expression of the *Slc25a1* (*p* ≤ .008), *Slc25a5* (*p* < .014), and *Slc25a10* (*p* < .015) genes encoding citrate transporter, adenine nucleotide translocator and decarboxylate transporter, respectively, was shown in aggressive mice and *Slc25a1* (*p* ≤ .026)—in defeated mice. The *Slc25a47* gene was up-regulated in both groups, in the winners (*p* < .0001, *q* < .005) and in the losers (*p* < .0001, *q* ≤ .005).

In the VTA (Fig. 1), the expression of the *Slc25a1* gene (*p* < .004) encoding the citrate transporter and the *Slc25a25* gene (*p* < .029) encoding the phosphate carrier were down-regulated in the losers *vs* the controls. In the striatum of the winners the *Slc25a46* gene was down-regulated (*p* ≤ .025) and the *Slc25a47* gene was up-regulated (*p* < .0001, *q* < .044).

As indicated by the data of transcriptome analysis, in the brain regions of defeated or aggressive male mice no changes in expression were observed for the *Slc25a2* and *Slc25a15* genes encoding ornithine carriers; the *Slc25a3, Slc25a4, Slc25a6,* and *Slc25a31* genes encoding inorganic phosphate (*PiC*) carriers; the *Slc25a7, Slc25a8, Slc25a9, Slc25a14* and *Slc25a27* genes encoding uncoupling proteins that serve as regulated proton channels or transporters; the *Slc25a23* and *Slc25a24* genes encoding Ca^2+^-sensitive mitochondrial carriers; the *Slc25a17* gene encoding the peroxisomal transporter; the *Slc25a20* gene encoding carnitine/acylcarnitine carriers; the *Slc25a21* gene encoding oxoadipate carrier; the *Slc25a26* gene encoding S-adenosylmethionine carrier; the *Slc25a30* gene encoding the carrier of many small metabolites; the *Slc25a33* and *Slc25a36* genes encoding pyrimidine nucleotide carriers; the *Slc25a32* gene *en*coding folate carrier; the *Slc25a37* gene encoding iron carriers, and the *Slc25a30*, *Slc25a34, Slc25a35, Slc25a43*, *Slc25a51*, and *Slc25a53* genes with unknown functions.

### Alternative splicing or exon skipping identified in Slc25* gene family

We applied rMATS software [47] to assess alternative splicing (AS) in Slc25* family genes in 5 brain regions based on RNA-Seq raw reads repository. Of 53 Slc25a* genes AS was detected for 26 genes, which changed their expression in defeated mice (Additional file 1: Table S3). The largest number of isoforms was observed for the *Slc25a1, Slc25a16, Slc25a19, Slc25a23, Slc25a26* and *Slc25a35* (5 AS events) and *Slc25a3, Slc25a22, Slc25a39, Slc25a46* genes (4 AS events).

In five brain regions we observed 98 exon skipping (ES) events encompassing 26 Slc25* genes and 45 distinct exons (Additional file 1: Table S3). Of those genes six genes manifest ES in all five brain regions (bold type); 4 genes manifest AS in 4 brain regions (bold italic) and 5 genes display AS in 3 brain regions. Six genes maintain 2 alternative exons (Additional file 1: Table S3; # alt exons). The skipping of the same exon (s) has been observed across corresponding brain regions (including genes presenting 2 ES events), thus increasing the confidence of non-random splicing events. For cross-validation we used ‘knownalt’ annotation table for alternative exons from the UCSC Genome Browser database [48], dated March 2016 (mm10 release). It comprises 12 Slc25* genes with alternative (cassette) exons annotated in the last column as ‘confirmed’ (Additional file 1: Table S3). We also used large scale alternative splicing annotation project presented in [45] comprising 38 Slc25* genes as alternatively spliced for ES events, since it identified far more AS events than those currently presented in Ref-Seq repository. We ascertained that all of our genes were indicated in the project [45] as displaying ES events.

### Principal component analysis of the expression of Slc25a* and Mrpl* and Mrps*-associated genes which changed their expression in affected male mice

**Fig. 2:**
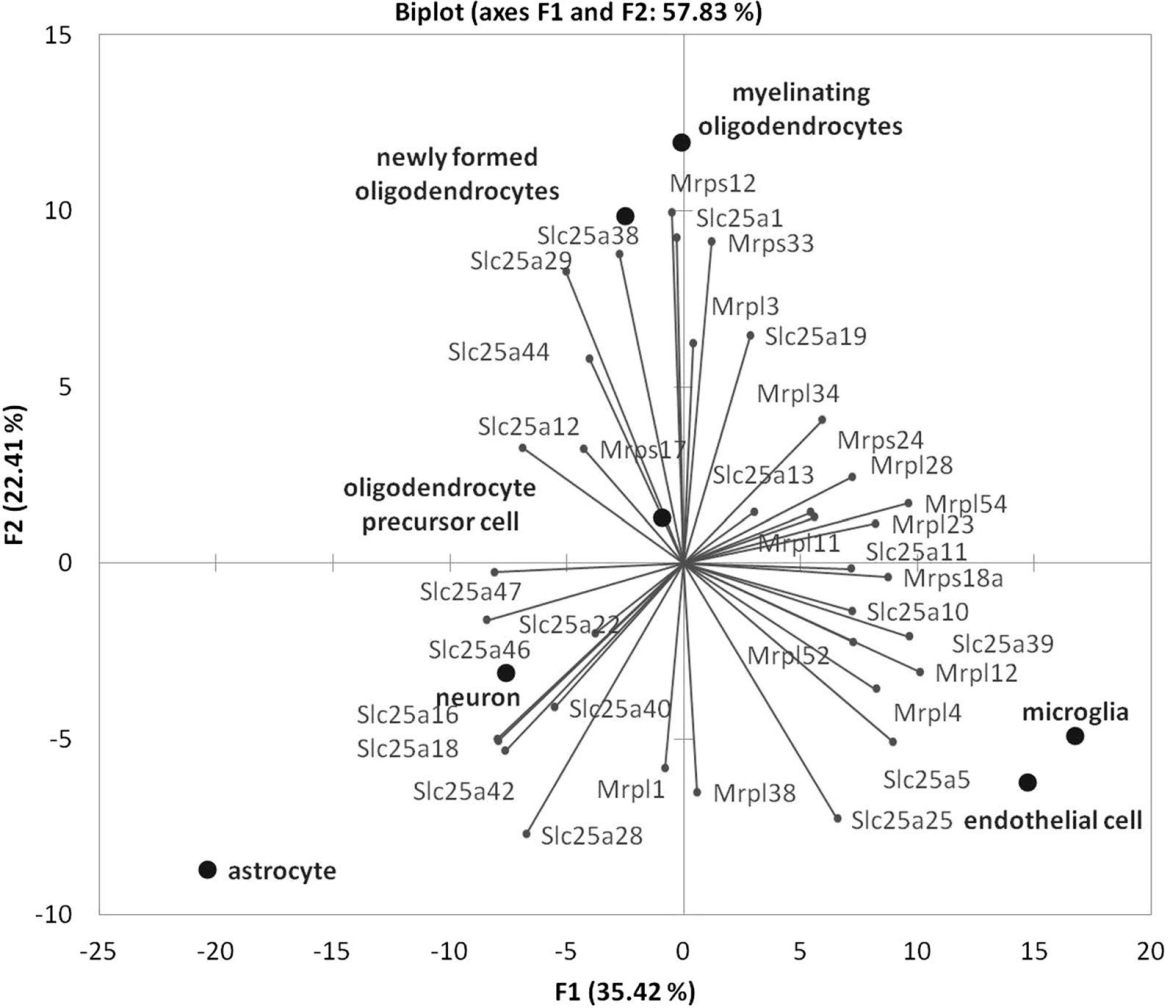
Principal component analysis plot based on the expression profiles in FPKM units, taken from [45] for illustration of brain cell type specific expression of particular Slc25a* genes

To assess the degree of cell lines-specific expression of genes of interest we performed PCA based on the co-variation of the Slc25a* and Mrpl* and Mrps* genes using the expression profiles of the samples comprised of RNA-Seq FPKM data for 7 types of brain specific cell lines [45]: astrocytes, neurons, oligodendrocyte precursor cells, newly formed oligodendrocytes, myelinating oligodendrocytes, microglia, and endothelialcells. PCA biplot presented in Fig. 2 underscores clustering of specific cell lines for one side, and for another, we see preferential genes expression for these cell line clusters. We observed compact distribution of the *Slc25a13* and *Slc25a19* together with the *Mrps33, Mrps24, Mrpl3, Mrpl5, Mrpl11, Mrpl23, Mrpl34,* and *Mrpl28* genes in myelinating oligodendrocytes and the *Slc25a10, Slc25a11, Slc25a39* genes with the *Mrps18a, Mrpl12, Mrpl4,* and *Mrpl52* genes in microglia. In newly formed oligodendrocytes the *Slc25a1, Slc25a29, Slc25a38,* and *Slc25a44* genes were found together with the *Mrps17* genes. Compact clustering of samples underscores distinct expression pattern for the genes under consideration in each type of cells.

### Correlation analysis

Pearson correlation analysis revealed significant correlations between FPKM parameters of the Slc25a* genes and the Mrpl* and Mrps* genes in different brain regions. Most correlations with mitoribosomal genes were shown for differentially expressed *Slc25a1, Slc25a10, Slc25a11, Slc25a28, Slc25a38,* and *Slc25a39* genes and a smaller number of correlations ˗ for the *Slc25a22, Slc25a40, Slc25a46,* and *Slc25a47* genes. The smallest number of significant correlations was found for the *Mrpl3, Mrpl23,* and *Mrpl52* genes (Additional file 2: Table S2). The FPKM values for most Slc25* and Mrp* genes in one cell (microglia, endothelial cells, neurons, oligodendrocytes) as a rule are significantly cross-correlated.

## Discussion

The repertoire of Slc25a* mitochondrial carrier family numbers 47 members. This is the largest number of transporter genes among all Slc* transporter families of 52 different subfamilies with important physiological and pathological functions [19, 40, 42].

### Differentially expressed genes in different brain regions of male mice with alternative social experience

The up-regulation of most Slc25a* genes in the hypothalamus of both depressive and aggressive mice may be a response to chronic social stress inducing the development of anxiety in both participants of social conflicts [49, 50]. Dysfunction of these genes implicates disturbances in citrate, glutamate, phosphate, decarboxylate etc. metabolism as well as energy metabolism. Expression of the genes of unknown function, *Slc25a40* and *Slc25a47* in the winners and *Slc25a40* and *Slc25a46* in the losers, was reduced specifically in this region. Earlier we found similar up-regulation of most mitochondrial ribosomal (Mrpl* and Mrps* families) genes in the hypothalamus of mice of both social groups [33, 38; Additional file 1: Table S2]. Therefore, it is natural to assume an elevated co-expression of numerous genes under chronic social stress of agonistic interactions.

In the hippocampus up-regulation of most Slc25a* genes is indicative of impaired glutamate and carnitine metabolism in mitochondria. The *Slc25a5* and *Slc25a13* genes encode adenine nucleotide translocator, the most plentiful protein in the inner mitochondrial membrane which exports ATP from the mitochondrial matrix and imports ADP into the matrix [51]. The adenine nucleotide translocators are important structural components of the mitochondrial permeability transition pore whose opening can lead to cell death through apoptosis or necrosis [52, 53]. Interestingly, these genes were oppositely regulated in the winners and losers. Decreased cell proliferation in defeated mice [54–56] and enhanced neurogenesis in aggressive mice [57] were previously shown in the dentate gyrus of the hippocampus. Therefore, it can be assumed that there is an association between altered neurogenesis and up- or down-regulation of Slc25a* transporter genes in male mice with alternative social experience.

The midbrain raphe nuclei contain the pericaryons of serotonergic neurons, which are involved in the regulation of many physiological, behavioral, and emotional processes. As shown earlier, repeated experience of aggression and defeats is accompanied by decreased serotonergic activity [28, 34] and down-regulation of serotonergic genes—*Tph2, Maoa, Slc6a4*, *Htr’s* [32, 36, 37] in this brain region. Thus, we can assume that decreased expression of some Slc25a* genes may be associated with decreased serotonergic activity in the midbrain raphe nuclei of aggressive and defeated mice.

In the VTA the expression of the *Slc25a1* gene, encoding the citrate transporter and the *Slc25a25* gene encoding the phosphate carrier were down-regulated in the losers only. In the striatum the *Slc25a46* gene was down-regulated and the *Slc25a47* gene was up-regulated only in the winners.

Little is known about the most recently discovered the *Slc25a46* and *Slc25a47* genes. Accordingly, literature on transport activities for these carriers remains scarce. Some interesting findings regarding relatively unknown proteins were obtained by a more comprehensive search. However, it is known that Slc25a*40-*47* genes are involved in a wide spectrum of neurological diseases and multiple neuropathies.

Interestingly, approximately ten of the Slc25a* genes in humans were considered earlier as housekeeping genes [58], which are typically essential genes that are expressed in all cells of an organism under normal and pathophysiological conditions and are required for the maintenance of basic cellular functions. Under repeated agonistic interactions expression of at least 7 of these genes—the *Slc25a5, Slc25a11, Slc25a28, Slc2538, Slc25a39, Slc25a44,* and *Slc25a46*—were changed in different brain regions of male mice. On the one hand, changes in expression of some Slc25a* genes may be specific for the mouse brain only. On the other hand, we can suggest that repeated agonistic interactions induce enormous changes in brain regulation forming psycho- and neuropathologies, which per se may impair the carrier function. The *Slc25a3, Slc25a26, Slc25a32* genes do not change their expression and can still be considered as housekeeping genes.

Previous data showed a brain region-specific bias of mitochondrial ribosomal genes—*Mrpl* and *Mrps*—in the aggressive and defeated mice [33, 38]. In this study we found a similar effect of agonistic interactions on specific functions of mitochondrial transporter family Slc25a* gene*s*. Our findings re-confirmed the development of mitochondrial dysfunction shown earlier, which was also confirmed by a high correlation rate between the expression of the Mrps* and Mrpl* genes and the Slc25a* genes in different brain regions. A significant correlation between the expression profiles of the Mrpl* genes and Slc25a* genes may indicate, on the one hand, that their functions interrelate and, on the other hand, that an altered expression of the Slc25a* genes may serve as a marker of mitochondrial dysfunctions in the brain regions.

According to Zhang et al. data [45] (Table 1), the *Slc25a1* gene is expressed in newly formed oligodendrocytes, the *Slc25a5* gene—in endothelial cells where it has significant correlation with expression of *Mrpl38* gene; the *Slc25a10* and *Slc25a11* genes are expressed in microglia; the *Slc25a12* gene—in oligodendrocyte precursor cells, the *Slc25a13* gene—in myelinating oligodendrocytes, the *Slc25a25* gene—in endothelial cells. Alternatively spliced transcript variants (2) have been observed for the *Slc25a13* gene. The *Slc25a18* gene is expressed in astrocytes and the *Slc25a22, Slc25a40,* and *Slc25a42* genes—in neurons. RNA-Seq method revealed 4 isoforms of the *Slc25a22* gene. The *Slc25a19* gene is expressed in myelinating oligodendrocytes, the *Slc25a47* gene—in oligodendrocytes precursor cells. The *Slc25a28* and *Slc25a46* genes are expressed in astrocytes. Alternatively spliced transcript variants have been found for the *Slc25a28* and *Slc25a29* genes. The *Slc25a29* and *Slc25a38* genes are expressed in newly formed oligodendrocytes.

**Table 1:** Distribution of differentially expressed *Mrps, Mrpl* genes and Slc25a* genes in nervous cells according to [45]

Cells [45]	Slc25a* genes	*Mrpl, Mrps genes* [33, 38]
Oligodendrocyte precursor cells	*Slc25a12, Slc25a47*	
Newly formed oligodendrocytes	*Slc25a1, Slc25a29, Slc25a38, Slc25a44*	*Mrps17*
Myelinating oligodendrocytes	*Slc25a13, Slc25a19*	*Mrps33, Mrps24, Mrpl3, Mrpl11, Mrpl23, Mrpl28, Mrpl34, Mrpl54*
Neurons	*Slc25a22, Slc25a40, Slc25a42*	*Mrpl1*
Microglia	*Slc25a10, Slc25a11, Slc25a39*	*Mrps18a, Mrpl12, Mrpl4, Mrpl52*
Endothelial cells	*Slc25a5, Slc25a25*	*Mrpl38*
Astrocytes	*Slc25a16, Slc25a18, Slc25a28, Slc25a46*	

Thus, our data indicate that the prominent role of mitochondria to produce the energy of the cell, ATP (i.e., phosphorylation of ADP) through respiration or regulation of cellular metabolism [59], involving the citric acid cycle or the Krebs cycle, may be disturbed under agonistic interactions leading to the development of psychoemotional disorders in male mice. It is also assumed non-fulfillment of other genomic functions of mitochondria in the processes of signaling, cellular differentiation, cell death, and maintenance of the cell cycle and growth [60]. Mitochondrial biogenesis, in turn, is temporally coordinated with these cellular processes [61, 62].

### Alternative splicing or exon skipping identified in Slc25* gene family

Based on phasing assessments in coding sequence exons we found that of 98 events 56 were ESs with the length not divisible by three, this way shifting the open reading frame unless compensated by another AS event, which is a rare occurrence, especially for non-adjacent exons, whereas the case of adjacent exons can be excluded by the identification method [47] except for mutually excluding exons (not identified). It implies that in the Slc25* family AS is extensively used, as is also the case for multicomponent complexes of ribosomal, chromatin remodeling, splicing machineries, for real time homeostasis maintenance by subjecting aberrant transcripts to NMD as the means of auto-/cross-regulation of expression, as has been reported previously [63]. Nevertheless, in each brain region we observed at least by two functional isoforms for the *Slc25a16, Slc25a19, Slc25a23, Slca25a26,* and *Scl25a35* genes. The most variable in terms of the number of isoforms were the *Slc25a23* and *Slc25qa26* genes (Additional file 1: Table S3; ‘#alt exons’), though some of the isoforms were NMD related.

Our data revealed numerous instances of alternative transcripts (Additional file 1: Table S3). The previous reports on AS of Slc25a* genes, in particular exon 9 skipping in the *Slc25a23, Slc25a24, Slc25a25* [64, 65], exons 5–11 skipping in the *Slc25a1*3 [66, 67] underscored the deleterious effects of the alternative isoforms except for the full length ones [65–67]. Notably, short isoforms of the *Slc25a13* gene were suggested as potential markers of citrin deficiency [67].

It is worth noting that skipping of the second/third exon of the *Slc25a3* gene identified also in our study was shown to be obligatory due to its evolutionary duplication, so that only one exon should be retained for making a functional transcript [68]. Consequently, the majority of isoforms detected for this gene were with exon skipping.

AS data from [45] comprised 189 unique ES events for the Slc25a* genes compared to our 45 ES events underlining a significantly higher reported coverage rate in [45]. They were classified as follows: 78 ES events were Nonsense Mediated Decay (NMD) inducing events (both with included and excluded exons) while ES events retaining coding potential were only 45 [45]. Events not specified for coding potential (UTR-related and noncoding RNA) numbered 59. Thus, the largest class proved to be NMD-related events.

While we haven’t witnessed the cases with drastic isoforms ‘switching’, there might as well be a slight shift of isoforms ratio sufficient to yield a pathological state provided that mitochondrial energy function is intense. The existence of region-specific mitochondrial transporter gene isoforms does not seem to affect the brain regions, but using a more precise RNA-Seq technology we were able to see consistent elevations and downturns of expression rate for particular groups of mitochondrial transporter proteins across the brain regions which were elucidated by PC analysis—probably by deleting this sequence.

Malacards database shows that most Slc25a* genes are involved in the development of numerous psychoemotional and neurological diseases. Changes in expression of the Slc25a* genes may be indicative of dysfunctions in the work of mitochondria and may be causes and consequences of such diseases. The *Slc25a1* gene is involved in a variety of cognitive and psychiatric disorders as well as in impaired neuromuscular disorders [69, 70]. Diseases associated with SLC25A5 protein include non-syndromic intellectual disability, Huntington’s and Parkinson’s diseases. The *Slc25a10* gene may be involved in some 1000 central nervous system diseases such as mood disorders, anxiety, depression and others. Polymorphisms in the *Slc25a12* gene may be associated with over 3000 diseases, and mutations in this gene may also be a cause of global cerebral hypomyelination. Diseases associated with SLC25A12 protein include epileptic encephalopathy, Asperger Syndrome, delayed cognitive and psychomotor development, psychomotor retardation. Mutations in the *Slc25a13* gene result in citrullinemia type II, which is characterized by neuropsychiatric symptoms including abnormal behaviors, loss of memory, seizures and coma. Inactivity of the Slc25a25 AGC1 in a patient could be associated with a mutation in a conserved glutamine residue. The pathological consequences of this mutation include severe hypotonia, halted psychomotor development and convulsions. The *Slc25a16* gene encodes a mitochondrial carrier associated with an autoimmune disease that results in hypothyroidism. Mutations in the *Slc25a22* gene are associated with early infantile epileptic encephalopathy and more than 800 glutamic acid related disorders such as major depression, bipolar disorder, psychosis, and motor neuron disease. A mutation in the *Slc25a19* gene was found to be associated with Amish microcephaly, neuropathy and bilateral striatal necrosis. The *Slc25a29* gene codes for a mitochondrial carrier, palmitoylcarnitine transporter, the clinical consequences of its alteration may lead to hypoglycaemia, hyperammonaemia, cardiomyopathy, liver failure, and encephalopathy. The *Slc25a38* gene, probably, is involved in the biosynthesis of heme. Diseases associated with the *Slc25a46* gene include hereditary motor and sensory neuropathy. Obviously, as a consequence, the development of depression is accompanied by changes in Slc25a* gene expression in different brain regions. The above data indicate that psychoneurological disorders, as a rule, are accompanied by mitochondrial dysfunctions, which may be at the root of metabolic changes involving numerous biochemical pathways.

Although it is well known that in treatment of depression monoaminergic antidepressants can improve cognitive and emotional performance, most antidepressants have limited clinical efficacy. It has been supposed that alterations in mitochondrial morphology, brain energy metabolism, and mitochondrial enzyme activity may be involved in the pathophysiology of different neuropsychiatric disorders such as depression, bipolar disorder, schizophrenia [11, 15, 17, 71]. We agree with Gardner and Boles [9] that understanding various concepts of mitochondrial dysfunction in pathogenesis of depression helps to generate novel targeted therapeutic approaches to depression treatment.

## Conclusions

We found experimentally that a mixed anxiety/depression-like state developing in animals under chronic social defeat stress is accompanied by a brain region-specific changes in the expression of mitochondrial Slc25a* transporter genes. Our findings reconfirmed the development of mitochondrial dysfunction shown earlier, which was also confirmed by a high correlation rate between the expression of mitochondrial Mrps* and Mrpl* genes and the Slc25a* genes in different brain regions. This correlation may indicate, on the one hand, that functions of these genes interrelate and, on the other hand, that an altered expression of the Slc25a* genes may serve as a marker of mitochondrial dysfunctions in brain.

## Additional files

**Additional file 1: Table S1.** Mitochondrial solute carrier Slc25* gene family with changed expression in brain regions under agonistic interactions in male mice. **Table S2.** Differentially expressed Mrp* genes in different brain regions of the winners and losers [33, 38]. **Table S3.** Exon skipping events observed in 5 brain regions for Slc25* gene family.

**Additional file 2: Table S1.** FPKM values for Slc25a* transcripts analyzed in the study. **Table S2.** Correlation matrix [Pearson (n − 1)] across 45 samples (all regions) between FPKM of differentially expressed Mrp* and Slc25a* genes.

## Abbreviations

PCA: principal component analysis
AHC: agglomerative hierarchical clustering
MDS: multidimentional scaling
AS: alternative splicing
ES: exon skipping
NMD: nonsense mediated decay
FPKM: fragments per kilobase of transcript per million mapped reads
