# Supplementary material for: Altered Slc25 family gene expression as markers of mitochondrial dysfunction in brain regions under experimental mixed anxiety/depression-like disorder
Source: BMC Neurosci. 2018 Dec 11;19:79. doi: 10.1186/s12868-018-0480-6 (PMC6288882; doi:10.1186/s12868-018-0480-6)
Supplement: Supplementary file 1 — Additional file 1: Table S1. Mitochondrial solute carrier Slc25* gene family with changed expression in brain regions under agonistic interactions in male mice. Table S2. Differentially expressed Mrp* genes in different brain regions of the winners and losers [33, 38]. Table S3. Exon skipping events observed in 5 brain regions for Slc25* gene family. [file 12868_2018_480_MOESM1_ESM.doc]

**Additional file 1. (Table S1–S3)**

| Table S1. Mitochondrial solute carrier family genes with changed expression in brain regions under agonistic interactions in male mice (according to DAVID, UniProt, Gene Card databases) | | | |
| --- | --- | --- | --- |
| Gene  symbol | Solute carrier family genes | Winners | Losers |
|  | **Hypothalamus** |  |  |
| *Slc25a1* | mitochondrial carrier, citrate transporter, member 1 |  | **Δ** |
| *Slc25a10* | [mitochondrial carrier, dicarboxylate transporter, member 10](https://david.ncifcrf.gov/geneReportFull.jsp?rowids=27376) | **Δ Δ** | **Δ Δ Δ Δ*** |
| *Slc25a11* | mitochondrial carrier, oxoglutarate transporter, member 11 |  | **Δ** |
| *Slc25a12* | mitochondrial carrier, Aralar, member 12 |  | **Δ** |
| *Slc25a16* | mitochondrial carrier, Graves disease autoantigen, member 16 |  | **▼** |
| *Slc25a18* | mitochondrial carrier, glutamate transporter, member 18 (GC) |  | **Δ Δ*** |
| *Slc25a19* | mitochondrial thiamine pyrophosphate carrier, member 19 |  | **Δ** |
| *Slc25a22* | [mitochondrial carrier, glutamate, member 22](https://david.ncifcrf.gov/geneReportFull.jsp?rowids=68267) | **Δ Δ Δ*** | **Δ Δ Δ Δ*** |
| *Slc25a25* | mitochondrial carrier, phosphate transporter, member 25 |  | **Δ Δ*** |
| *Slc25a28* | mitochondrial carrier, [iron](http://pathcards.genecards.org/card/mitochondrial_iron-sulfur_cluster_biogenesis) transporter, [member 28](https://david.ncifcrf.gov/geneReportFull.jsp?rowids=246696) (GC) |  | **Δ Δ*** |
| *Slc25a29* | mitochondrial carrier, palmitoylcarnitine transporter, member 29 | **Δ Δ** | **Δ Δ Δ*** |
| *Slc25a38* | mitochondrial glycine transporter [**member 39**](https://david.ncifcrf.gov/geneReportFull.jsp?rowids=68066) | **Δ Δ Δ Δ*** | **Δ Δ Δ Δ*** |
| *Slc25a39* | transport amino acids [member 39](https://david.ncifcrf.gov/geneReportFull.jsp?rowids=68066)(GC) |  | **Δ** |
| *Slc25a40* | mitochondrial carrier ornithine transporter, [member 40](https://david.ncifcrf.gov/geneReportFull.jsp?rowids=319653) (UP) | **▼▼** | **▼▼▼*** |
| *Slc25a42* | mitochondrial carrier, coenzyme A transporter [**member 42**](https://david.ncifcrf.gov/geneReportFull.jsp?rowids=73095) (UP) |  | **Δ** |
| *Slc25a44* | [member 44](https://david.ncifcrf.gov/geneReportFull.jsp?rowids=229517) |  | **Δ** |
| *Slc25a46* | member 46 |  | **▼▼*** |
| *Slc25a47* | member 47 | **▼▼▼▼*** |  |
|  | **Hippocampus** |  |  |
| *Slc25a5* | mitochondrial carrier, adenine nucleotide translocator, member 5 | **Δ** |  |
| *Slc25a13* | mitochondrial carrier, adenine nucleotide translocator, member 13 |  | **▼▼** |
| *Slc25a22* | mitochondrial carrier, glutamate, member 22 |  | **Δ** |
| *Slc25a28* | mitochondrial carrier,  [iron](http://pathcards.genecards.org/card/mitochondrial_iron-sulfur_cluster_biogenesis) transporter, [member 28](https://david.ncifcrf.gov/geneReportFull.jsp?rowids=246696) (GC) |  | **Δ** |
| *Slc25a29* | mitochondrial carrier, palmitoylcarnitine transporter, member 29 |  | **Δ** |
| *Slc25a40* | [member 40](https://david.ncifcrf.gov/geneReportFull.jsp?rowids=319653) |  | **▼** |
| *Slc25a42* | [member 42](https://david.ncifcrf.gov/geneReportFull.jsp?rowids=73095) |  | **Δ** |
| *Slc25a44* | [member 44](https://david.ncifcrf.gov/geneReportFull.jsp?rowids=229517) |  | **Δ** |
|  | **Midbrain raphe nuclei** |  |  |
| *Slc25a1* | [mitochondrial carrier, citrate transporter, member 1](https://david.ncifcrf.gov/geneReportFull.jsp?rowids=13358) | **▼▼** | **▼** |
| *Slc25a5* | [mitochondrial carrier, adenine nucleotide translocator, member 5](https://david.ncifcrf.gov/geneReportFull.jsp?rowids=11740) | **▼** |  |
| *Slc25a10* | [mitochondrial carrier, dicarboxylate transporter, member 10](https://david.ncifcrf.gov/geneReportFull.jsp?rowids=27376) | **▼** |  |
| *Slc25a47* | member 47 | **Δ Δ Δ Δ*** | **Δ Δ Δ Δ*** |
|  | **Striatum** |  |  |
| *Slc25a46* | [member 46](https://david.ncifcrf.gov/geneReportFull.jsp?rowids=67453) | **▼** |  |
| *Slc25a47* | [member 47](https://david.ncifcrf.gov/geneReportFull.jsp?rowids=104910) | **Δ Δ Δ Δ*** |  |
|  | **VTA** |  |  |
| *Slc25a1* | mitochondrial carrier, citrate transporter, member 1 |  | **▼▼** |
| *Slc25a25* | [mitochondrial carrier, phosphate carrier, member 25](https://david.ncifcrf.gov/geneReportFull.jsp?rowids=227731) |  | **▼** |
| Note:▼ - downregulation;  **Δ** - upregulation genes expression in comparison with the controls; ▲ - p ≤ 0.05; ▲▲ - *P* ≤ 0.01; ▲▲▲ -  *P* ≤ 0.001; ▲▲▲▲ - *P* ≤ 0.0001; **Δ** - p ≤ 0.05;  **ΔΔ** - *P* ≤ 0.01;  **ΔΔΔ** -  *P* ≤ 0.001;  **ΔΔΔΔ** - *P* ≤ 0.0001;* - q < 0.05. VTA – ventral tegmental area | | | |

| Table S2. Differentially expressed *Mrp** genes in different brain regions of the winners and losers [33,38] | | | |
| --- | --- | --- | --- |
| **Winners** |  | **Losers** |  |
| **Midbrain raphe nuclei** | | | |
| *Mrpl28, Mrpl23*  *Mrps17, Mrps24* | down  down |  |  |
| **Hippocampus** | | | |
| *Mrps33* | up |  |  |
| **Hypothalamus** | | | |
|  |  | *Mrps18a, Mrps12* | up |
| ***Mrpl54, Mrpl4, Mrpl38, Mrpl28,*** *Mrpl11* | up | ***Mrpl54, Mrpl4, Mrpl38, Mrpl28,*** *Mrpl12, Mrpl52*  *Mrpl23,Mrpl34,Mrpl1,Mrpl3* | up  down |

| Table S3. Exon skipping events observed in 5 brain regions for *Slc25** gene family | | | | | | | | |
| --- | --- | --- | --- | --- | --- | --- | --- | --- |
|  | gene_id | HPC | HPT | STR | MRN | VTA | #alt_exons | Status by Refgene |
| 1 | Slc25a1 | + | + | + | + | + | 1 | novel |
| 2 | Slc25a10 |  |  |  | + | + | 1 | novel |
| 3 | Slc25a11 |  |  | + | + |  | 1 | novel |
| 4 | Slc25a12 |  | + | + |  | + | 1 | novel |
| 5 | Slc25a14 |  | + | + | + |  | 1 | confirmed |
| 6 | **Slc25a16** | **+** | **+** | **+** | **+** | **+** | 2 | novel |
| 7 | Slc25a17 |  | + |  |  |  | 1 | novel |
| 8 | Slc25a18 |  |  | + | + | + | 1 | novel |
| 9 | **Slc25a19** | **+** | **+** | **+** | **+** | **+** | 2 | confirmed |
| 10 | Slc25a22 |  | + | + | + | + | 1 | novel |
| 11 | **Slc25a23** | **+** | **+** | **+** | **+** | **+** | 3 | novel |
| 12 | **Slc25a26** | **+** | **+** | **+** | **+** | **+** | 5 | confirmed |
| 13 | Slc25a28 |  | + |  |  | + | 1 | confirmed |
| 14 | Slc25a29 |  |  |  |  | + | 1 | novel |
| 15 | Slc25a3 | + |  | + | + | + | 2 | novel |
| 16 | Slc25a30 | + |  | + |  |  | 1 | novel |
| 17 | Slc25a33 |  |  | + | + | + | 2 | novel |
| 18 | **Slc25a35** | **+** | **+** | **+** | **+** | **+** | 1 | confirmed |
| 19 | Slc25a36 |  | + |  | + |  | 1 | novel |
| 20 | Slc25a39 | + | + | + |  | + | 3 | novel |
| 21 | Slc25a42 | + |  |  |  | + | 1 | novel |
| 22 | Slc25a43 |  | + |  |  |  | 1 | confirmed |
| 23 | Slc25a46 | + | + | + | + |  | 4 | novel |
| 24 | Slc25a47 |  |  | + | + |  | 1 | novel |
| 25 | Slc25a51 |  | + |  | + | + | 1 | novel |
| 26 | Slc25a53 |  |  | + |  |  | 1 | novel |
| Note: ‘+’ sign denotes presence of the ES events. Genes with ES events that were present in all brain regions are bold typed. HPC – hippocampus, HPT - hypothalamus; STR – striatum; MRN – midbrain raphe nuclei; VTA – ventral tegmental area. | | | | | | | | |
